# Extragastrointestinal stromal tumor of the inferior vena cava: a case report

**DOI:** 10.1186/s40792-017-0329-8

**Published:** 2017-04-19

**Authors:** Kazuhide Ko, Kimiyoshi Shimanuki, Wataru Sakamoto, Keisuke Hara, Eiji Uchida

**Affiliations:** 1 Department of Surgery, Aizu Chuo Hospital, 1-1 Turugamachi, Aizuwakamatsu-shi, Fukushima Japan; 2 Department of Gastrointestinal and Hepatobiliary-Pancreatic Surgery, Nippon Medical School, 1-1-5 Sendagi, Bunkyo-ku, Tokyo, Japan

**Keywords:** Extragastrointestinal stromal tumor, c-kit-positive tumor, Tumor of the inferior vena cava

## Abstract

Here, we present a case report of a 60-year-old female with a 5-cm tumor in the inferior vena cava (IVC) that was positive for c-kit and CD34 expression. Thus, we considered this to be an extragastrointestinal c-kit-positive stromal tumor (EGIST). To the best of our knowledge, no primary EGISTs of the IVC have been described thus far. The potential occurrence of EGISTs outside the tubular gastrointestinal tract should be recognized in the differential diagnosis of tumors of the great vessels. Thus, we concluded that primary c-kit-positive stromal tumors of the IVC do indeed occur.

## Background

Tumors of the great vessels are rare, and the most common site of origin is the inferior vena cava (IVC). Although leiomyosarcoma (LMS) is the most common malignant tumor of the IVC, thus far, there has been no report of an extragastrointestinal c-kit-positive stromal tumor (EGIST) arising from the IVC. EGIST is a unique tumor that occurs outside the gastrointestinal tract with a positive c-kit expression and histological appearance similar to that of gastrointestinal stromal tumors (GISTs) [1, 2]. Here, we report on a case of IVC tumor positive for c-kit and CD34 expression which was considered EGIST.

## Case presentation

**Fig. 1:**
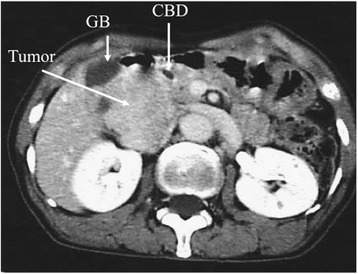
Abdominal computed tomography. Contrast-enhanced abdominal computed tomography demonstrated a large heterogeneous mass, 5 cm in length that was compressing the IVC and head of the pancreas. *GB* gall bladder, *CBD* common bile duct, *IVC* inferior vena cava

**Fig. 2:**
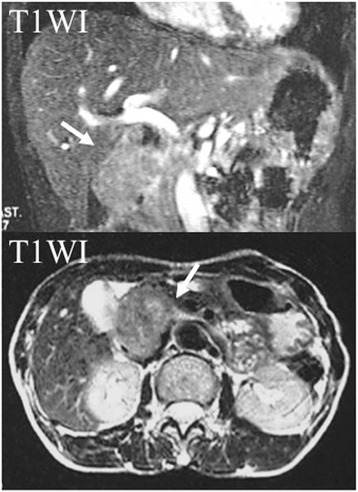
Abdominal magnetic resonance imaging. The *arrow* indicates the tumor. Magnetic resonance imaging shows a well-defined lesion along the course of the IVC. *IVC* inferior vena cava

**Fig. 3:**
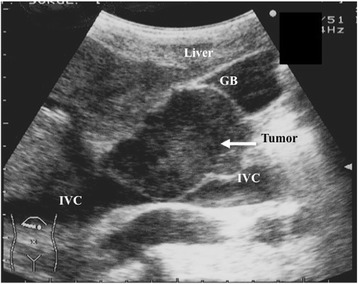
Abdominal ultrasonography. Abdominal ultrasonography revealed an abnormal mass in the infrahepatic portion, which compressed the gall bladder and liver. *GB* gall bladder, *IVC* inferior vena cava

A 60-year-old female presented with a 1-year history of vague right-sided abdominal pain. She had no weight loss or swelling in the legs. An abdominal mass in the right upper quadrant was palpated on physical examination. There were no abnormal laboratory findings. Upper gastrointestinal tract examination was normal except for the displacement of the duodenal loop by the abdominal mass. Colonoscopy and barium enema study findings were normal. Contrast-enhanced abdominal computed tomography demonstrated a large heterogeneous mass, 5 cm in length, with focal enhancement that markedly expanded the extraluminal site of the IVC and infrahepatic portion, which anteriorly displaced the bowel loops and compressed the IVC and head of the pancreas (Fig. 1). Magnetic resonance imaging indicated a well-defined lesion along the course of the IVC and in the infrahepatic portion. Its superior extent was clearly demonstrated, which did not cross the diaphragm but compressed the normal liver parenchyma (Fig. 2). Magnetic resonance cholangiopancreatography showed no abnormal findings in the pancreatic or common bile ducts. Abdominal ultrasonography revealed an abnormal mass in the infrahepatic portion, which compressed the gall bladder and liver. It was suspected that the tumor had partial continuity with the anterior wall of the IVC. The intraluminal surface of the IVC was smooth. There was no continuity with an organ of circumference (Fig. 3).

**Fig. 4:**
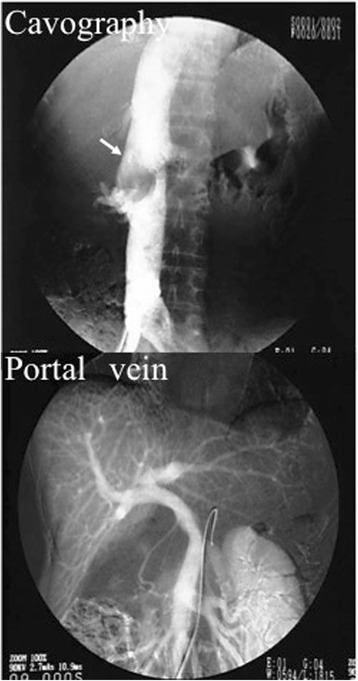
Abdominal angiography. The *arrow* indicates the compression site by the tumor. An inferior vena cavogram showed partial compression of the IVC. Superior mesenteric arteriography showed displacement of the portal vein. *IVC* inferior vena cava

**Fig. 5:**
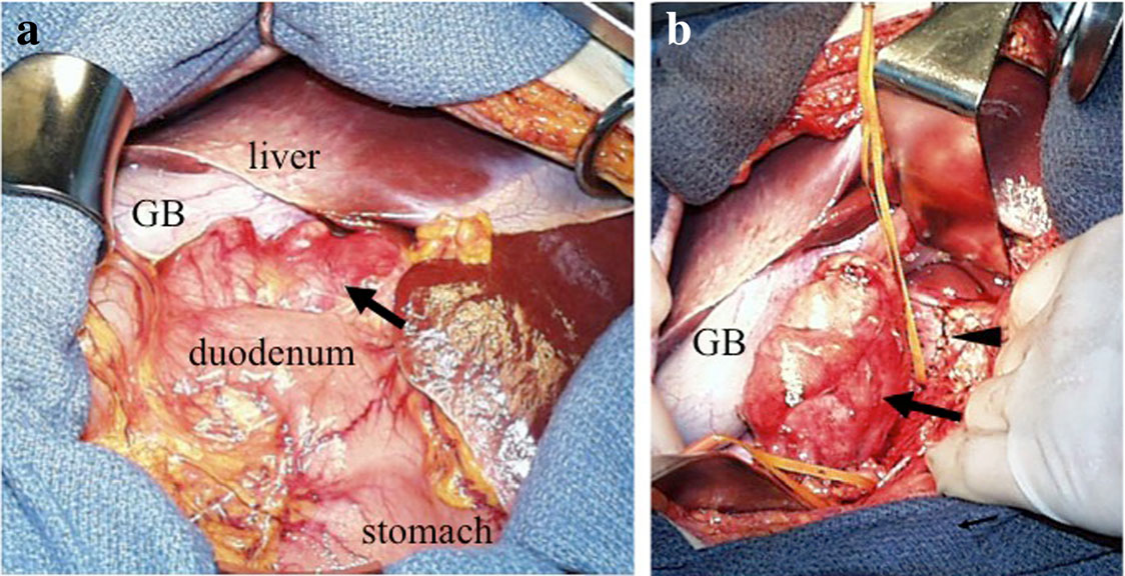
Operative findings. The *arrow* shows the tumor, and the *arrowhead* shows the IVC. A large retroperitoneal tumor was found located on the IVC. *GB* gall bladder, *IVC* inferior vena cava

It was suspected that this abnormal mass arose from extrahepatic, extrapancreatic, and extragastrointestinal origin. Thus, abdominal angiography was performed to detect the feeding artery of the tumor. Celiac and renal angiography demonstrated no tumor with neovascularity. Superior mesenteric arteriography showed displacement of the portal vein. An inferior vena cavogram showed partial compression of the IVC without collateral communication (Fig. 4). Preoperative ultrasonography-guided transcutaneous core needle biopsy was performed. Immunohistochemical staining of the biopsy specimen using the c-kit antibody displayed diffuse cytoplasmic staining, and the tumor cells were positive for CD34 expression. Based on the abovementioned examination findings, preoperative diagnosis was GIST in the retro-peritoneum and outside the gastrointestinal tract. During laparotomy, a large retroperitoneal elastic tumor was found on the IVC. We diagnosed it as EGIST arising from the IVC because there was no continuity with surrounding organs except the IVC. Because the tumor developed in the extraluminal site of the IVC, we confirmed that the lumen had not become narrow by applying the vascular forceps to the margin of the tumor. We then performed wedge resection of the IVC and continuous suture closure. The patient was treated by surgical resection without synthetic graft replacement of the IVC (Fig. 5).

**Fig. 6:**
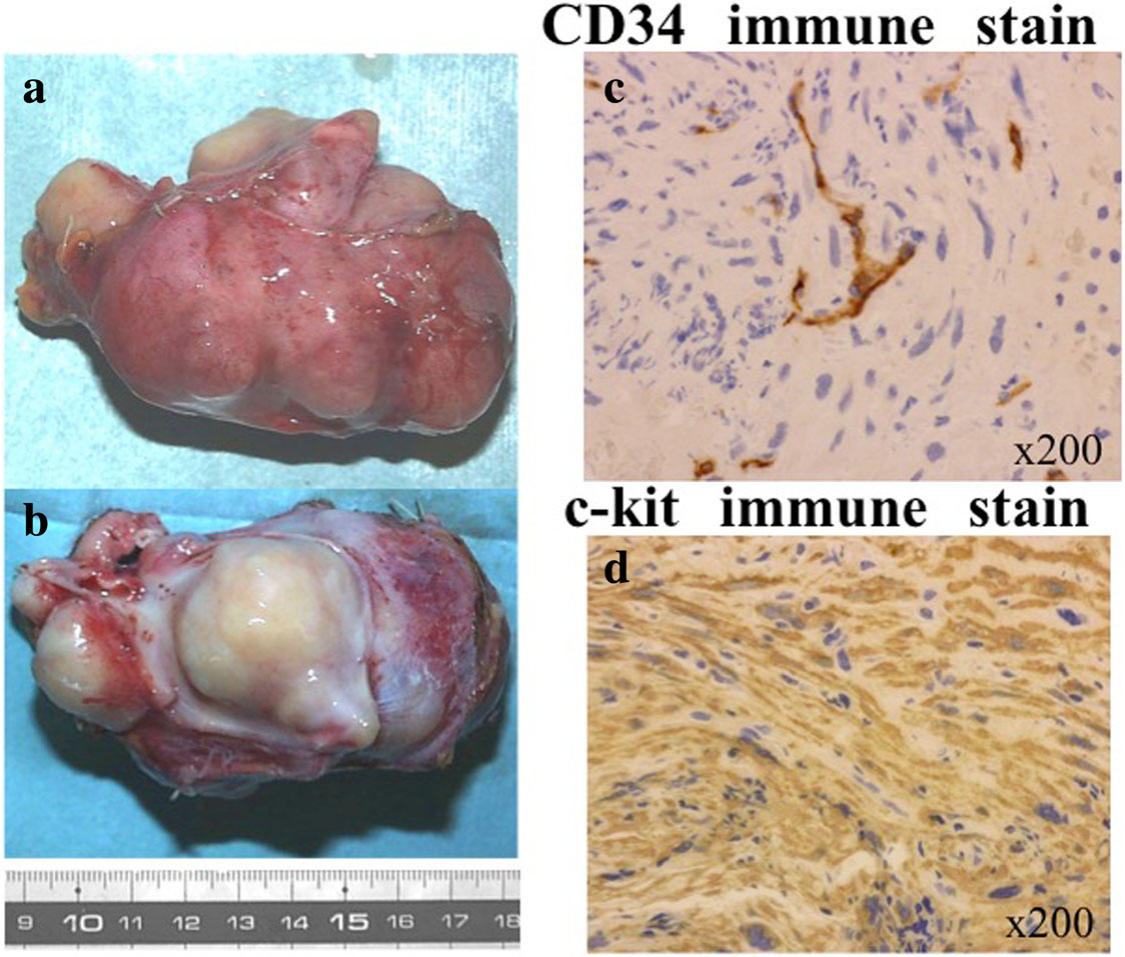
Gross pathology and immunohistochemical findings. A well-circumscribed and pseudo-capsulated white yellowish elastic mass (**a**), continued with the IVC wall, was observed to have a smooth intraluminal caval surface (**b**). Immunohistochemical staining demonstrated the tumor cells to be positive for CD34 (**c**) and c-kit (**d**) expression. *IVC* inferior vena cava

The well-circumscribed and pseudo-capsulated white yellowish elastic mass, which was continuous with the wall of the IVC, had a smooth intraluminal caval surface. The excision margin was observed to be free of the tumor (Fig. 6a, b). No hemorrhagic or necrotic lesions were noted on taking a cross section of the tumor.

**Fig. 7:**
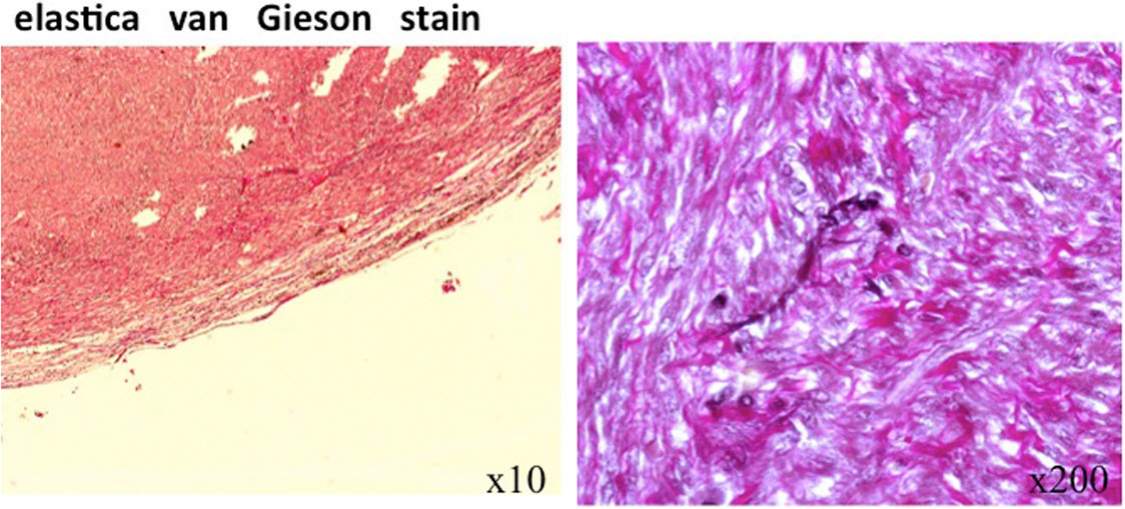
Elastica van Gieson stain. Interrupted elastic fiber of the IVC was shown, although there was continuity of the elastic fiber in the outer layer of this tumor as determined by elastica van Gieson staining. *IVC* inferior vena cava

Variable-sized smooth muscle cells were demonstrated in a whirling pattern (HE staining). In our case, there was low cellularity, minimal nuclear pleomorphism, absence of necrosis, and absence of an infiltrative growth pattern, and mitosis was under 1 per 30–50/high-powered field image (×400). Immunohistochemical staining demonstrated that tumor cells were positive for c-kit and CD34 expression (Fig. 6c, d). The tumor cells were negative for the S-100 protein. The histological features and staining pattern were consistent with a GIST. Interrupted elastic fiber of the IVC was shown, although there was continuity of the elastic fiber in the outer layer of this tumor as determined via elastica van Gieson stain (Fig. 7). The immunohistochemical profile, including c-kit and CD34 co-expression and absence of desmin and actin, specifically identified this tumor as GIST outside the tubular gastrointestinal tract. Based on these findings, we diagnosed that this tumor was EGIST originating from the smooth muscle in the media of the IVC wall. The postoperative course was uneventful, and there was no sign of recurrence.

## Discussion

GISTs were previously thought to be smooth muscle neoplasms, and most were classified as LMS. With the advent of immunohistochemistry and electron microscopy, it has become apparent that GISTs may have myogenic features, neural attributes, and characteristics of both muscle and nerve or may lack differentiation [3]. Sarlomo-Rikala et al. [4] pointed out that the c-kit antibody is a useful marker for diagnosing GISTs and for distinguishing them from true leiomyomas and neurogenic tumors. c-kit over expression because of activating mutations appears to drive the neoplastic growth of GISTs [5–7]. GISTs most commonly occur in the stomach (52%), small intestine (25%), esophagus (5%), and large bowel (11%) [8]. The most intriguing findings in the current study were the observation of a lesion that was located outside the gastrointestinal tract, primarily the mesentery, omentum, and retroperitoneum [1, 2], but fulfilled the histologic and immunohistochemical criteria for classification as GIST. EGIST is positive for c-kit expression, with histological appearance similar to GIST.

It is recognized that GIST cells exhibit characteristics similar to those of the interstitial cells of Cajal (ICC), the pacemaker cells of the gastrointestinal tract [9, 10]. Presence of an ICC system was reported in extragastrointestinal locations, guinea pig urinary bladder [11], guinea pig mesenteric arteries [12], sheep mesenteric lymphatic vessels [13], and fetal endothelial cells [14]. Bolton et al. identified non-contractile cells closely resembling ICC in the wall of the portal vein and mesenteric artery using immunohistochemical approaches in combination with confocal imaging [15]. Povstyan et al reported that two layers of ICC were detected by c-kit and methylene blue staining in the media of the rabbit portal vein in sub-endothelial intramuscular and deeper intramuscular positions [16]. As per these reports, c-kit-positive stromal tumors can occur in extragastrointestinal anatomic sites, particularly in those that are embryologically linked to mesenchymal cells, similar to the vessels. In our resected specimen, immunohistochemical staining could not demonstrate the normal media wall of the IVC to be positive for c-kit expression. However, it was confirmed that there was a vascular tumor of ICC origin. EGIST of the IVC is a very rare kind of tumor, and there are no reports of EGIST arising from the IVC till date.

Tumors of the IVC are uncommon malignancies with a tendency for both local recurrence and systemic dissemination. Surgical resection remains the therapeutic gold standard for GISTs and LMS, and tumor resectability is an important prognostic factor. However, LMS arising in the intima has poorer prognosis than mural sarcomas because intimal origin is a source of widespread metastases. Additionally, seeding of the tumor to distant sites occurs earlier. In our case, adjuvant therapy was not administered because there was no evidence of metastases and recurrences and the tumor was originating from the media of the vessel wall. Although distinction between GIST and LMS is important, GIST might be effectively treated with tyrosine kinase inhibitors, such as imatinib mesylate [17, 18]. There is the possibility that GIST occurred in the present case, which was initially reported as LMS of the IVC because those were histologically classified together as LMS owing to their similarities as determined via light microscopy. The potential occurrence of GIST-like tumors outside the gastrointestinal tract should be recognized in the differential diagnosis of tumors of the great vessels.

## Conclusions

We concluded that primary EGISTs of the IVC indeed occurred. However, the ultimate elucidation of the origin of vascular tumors resembling the tumor presented herein warrants future discovery and study of more vascular EGISTs.
